# Refining clinically relevant parameters for mis-splicing risk in shortened introns with donor-to-branchpoint space constraint

**DOI:** 10.1038/s41431-024-01632-9

**Published:** 2024-05-27

**Authors:** Katharine Y. Zhang, Himanshu Joshi, Rhett G. Marchant, Samantha J. Bryen, Ruebena Dawes, Michaela Yuen, Sandra T. Cooper, Frances J. Evesson

**Affiliations:** 1 Kids Neuroscience Centre, Kids Research, The Children’s Hospital at Westmead, Westmead, NSW Australia; 2 Functional Neuromics, Children’s Medical Research Institute, Westmead, NSW Australia; 3 School of Medical Sciences, Faculty of Medicine and Health, The University of Sydney, Sydney, NSW Australia; 4 Discipline of Child and Adolescent Health, Faculty of Medicine and Health, The University of Sydney, Westmead, NSW Australia; 5 Present Address: Centre for Population Genomics, Garvan Institute of Medical Research, UNSW & Murdoch Children’s Research Institute, Sydney & Melbourne, Australia; 6 Present Address: Big Data Institute and Centre for Human Genetics, University of Oxford, Oxford, UK; 7 Present Address: School of Biotechnology and Biomolecular Sciences, University of New South Wales, Sydney, Australia

**Keywords:** RNA splicing, Medical genetics

## Abstract

Intronic deletions that critically shorten donor-to-branchpoint (D-BP) distance of a precursor mRNA impose biophysical space constraint on assembly of the U1/U2 spliceosomal complex, leading to canonical splicing failure. Here we use a series of β-globin (*HBB*) gene constructs with intron 1 deletions to define D-BP lengths that present low/no risk of mis-splicing and lengths which are critically short and likely elicit clinically relevant mis-splicing. We extend our previous observation in *EMD* intron 5 of 46 nt as the minimal productive D-BP length, demonstrating spliceosome assembly constraint persists at D-BP lengths of 47-56 nt. We exploit the common *HBB* exon 1 β-thalassemia variant that strengthens a cryptic donor (NM_000518.5(HBB):c.79G > A) to provide a simple barometer for the earliest signs of space constraint, via cryptic donor activation. For clinical evaluation of intronic deletions, we assert D-BP lengths > 60 nt present low mis-splicing risk while space constraint increases exponentially with D-BP lengths < 55 nt, with critical risk and profound splicing abnormalities with D-BP lengths < 50 nt.

## Introduction

Precursor mRNA splicing to remove introns and join exons, producing mature mRNA, is a key regulatory step for gene expression. Canonical pre-mRNA splicing of introns is catalysed by a large complex of ribonucleoproteins known as the major spliceosome, which dynamically assembles on specific features of the intron. Sequential snRNP binding and transesterification reactions at the 5’ donor splice site (by the U1 snRNP) and the intronic branchpoint nucleotide (by U2) arranges the intron into a lariat structure and position the donor in proximity to the acceptor through formation of the B complex (consisting of U2, U4/U6 and associated factors), facilitating joining of the two exons (for detailed description of spliceosome structure and function, see [1]). Failure at any step of this sequential process can cause pre-mRNA splicing anomalies, such as exon skipping, cryptic splice site activation or intron retention. While the donor (GT) and acceptor (AG) consensus splice sites are largely invariant, there is a greater degree of flexibility in the branchpoint nucleotide and its surrounding features [2]. As such, many pre-mRNA splicing prediction algorithms can accurately predict the donor and acceptor sites and assess the effects of variants in these locations on pre-mRNA splicing. However, the effect of intronic sequence variants outside consensus splice sites - including branchpoint variants, cryptic splice sites, and deletions reducing intron length - remain overlooked by many informatics tools with significant implications for the provision of clinical genetic disease diagnoses.

Previously, we reported aberrant pre-mRNA splicing events caused by deletions in *DOK7* intron 1 and *EMD* intron 5 that spared consensus splice sites but reduced donor-to-branchpoint (D-BP) distance [3]. In both cases, splicing algorithms failed to predict any risk for mis-splicing, while functional studies on patient-derived samples demonstrated absence of canonical pre-mRNA splicing and deficiency of protein expression. In vitro spliceosome assembly studies revealed the pathogenic mechanism underpinning critical intronic shortening was an inability of the spliceosome to transition from A to B complexes, resulting in splicing failure. In-depth studies of an *EMD* whole gene construct established 45-47 nt as the threshold minimal D-BP distance enabling some canonical splicing and protein expression.

Short introns are rare in the human genome [3], suggestive of negative selective pressure, and resulting in a relative paucity of data for the accurate training of predictive algorithms compared to longer introns. While a subset of short introns with specific sequence features are spliced via an alternative mechanism [4], a significant proportion of short intron pre-mRNA splicing is still facilitated by the major spliceosome [5]. Assembly of the major spliceosome on introns involves the formation of a 17 nt RNA helix between the U6 and nucleotides near the acceptor, and a 14 nt helix between the U2 and branchpoint. As measured by a 3D cryo-EM structure of the assembled spliceosomal B complex, a minimum of ~21 nt is required to bridge the 15 nm space between these two bound components [6]. This approximate 52 nt lower limit for donor-to-branchpoint distance can be further reduced by movement of the U2 relative to the rest of the spliceosome, allowing for inefficient but achievable splicing of introns with a slightly shortened intron-to-branchpoint distance [3].

Previous studies of pre-mRNA splicing of the β-globin gene (*HBB*) using rabbit *HBB* implied the necessity of a minimal intron length for splicing, without defining the specific parameters or minimal length [7]. Variants in *HBB* leading to mis-splicing events are a common cause of human disease; a large proportion of β-thalassemia cases in Southeast Asia are caused by a NM_000518.5(HBB):c.79 G > A variant [8], which promotes the use of an alternate upstream cryptic splice site resulting in introduction of a premature termination codon [9]. Here, we use *HBB* as a model to refine minimal D-BP lengths across genomic contexts; the small size of *HBB* facilitates modelling of the entire gene in an expression construct for pre-mRNA splicing studies. The longer native *HBB* intron 1 length (130 nt, NM_000518.5) allows us to define saturating D-BP lengths conferring maximal splicing efficiency, not previously determinable for *EMD* intron 5 [3] due to its short, natural length (79 nt NM_000117.3). Our additional, deep empirical splicing data using *HBB* intron-1 now allows us to define: (a) the minimal D-BP length associated with a significant impairment in canonical splicing; (b) the D-BP length associated with no/minimal impact to canonical splicing and (c) the extent of any variation in splicing impairment at different D-BP lengths between *HBB* intron-1 and *EMD* intron-5 due to differences in nucleotide composition and genomic context; to inform (d) guidelines for clinical interpretation of mis-splicing risk from intronic deletions reducing D-BP length.

## Materials and Methods

### In silico prediction of branchpoints and effects of deletions in HBB intron 1

Potential branchpoints in *HBB* intron 1 were predicted using the Branchpointer algorithm [10]. Altered *HBB* transcript and intron 1 constructs were processed via SpliceAI [11] and BPP [12] respectively to identify predicted impact on *HBB* intron 1 splicing and branchpoint usage.

### HBB expression constructs

gBlock gene fragments (Integrated DNA Technologies, Singapore) containing FLAG-tagged *HBB* (genomic locus GRCh38.p14 11:5225464-5227071) were cloned into a pCMV6-entry vector (Origene, Rockville, MD, USA) via restriction digestion and ligation. Constructs were verified by Sanger sequencing (AGRF, Melbourne, Australia).

### Cell culture and transfection

COS-7 and HEK293 cells were cultured in Dulbecco’s Modified Eagle Medium (Thermo Fisher Scientific, Waltham, MA, USA) containing 10% Hyclone foetal bovine serum (GE Healthcare Life Sciences, Chicago, IL, USA) and 50 µg/mL gentamycin (Thermo Fisher Scientific). For *HBB* minigene transfection, COS-7 cells were seeded into 6-well plates and transfected 24 hours later with 6 µL Lipofectamine LTX (Thermo Fisher Scientific) and 3 µg plasmid DNA per well according to manufacturer’s instructions. Cells were harvested 48 hours post-transfection for protein and RNA analyses.

### Western Blotting

Cell pellets were harvested, lysed and a western blot was performed as previously described [13]. Primary antibodies used were anti-FLAG (epitope: DYKDDDDK, 1:2000, developed by Alfandari, D. from the University of Massachusetts, Amherst; obtained from the Developmental Studies Hybridoma Bank, created by the NICHD of the NIH and maintained at The University of Iowa, Department of Biology, Iowa City, IA, USA), anti-neomycin phosphotransferase (polyclonal, 1:1000, Sigma-Aldrich, Burlington, MA, USA). Fluorescent secondary antibodies used were anti-mouse IRDye 800CW and anti-rabbit IRDye 680CW (1:15,000, LI-COR Lincoln, NE, USA). Fluorescent blots were detected and imaged on the Odyssey XF system (LI-COR). Neomycin phosphotransferase (NPT) served as a control for transfection levels; the FLAG signal was normalised to NPT levels for quantification.

### RT-PCR and Sanger sequencing analysis

RNA was extracted from harvested cells using the RNEasy PLUS mini kit (Qiagen, Hilden, Germany), with an additional on-column DNAse digestion step using RNAse-free DNA (Qiagen), according to manufacturer’s instructions. cDNA was synthesised from 500 ng total RNA using the Superscript IV First-Strand Synthesis System (Thermo Fisher Scientific). PCR was carried out using MyTaq (Meridian Bioscience, Memphis, TN, USA) with primers listed in Table 1. All reactions were amplified using a forward primer which binds to the FLAG-tag within exon 1 of the *HBB* minigene (Exon1F), which excludes amplification from endogenous *HBB*. Cycling conditions were 95 °C for 5 minutes, 30 cycles 95 °C 20 seconds, 60–64 °C 15 seconds, 72 °C 20 seconds, then 72 °C 7 minutes. PCR products were run on 2% agarose gels in TAE buffer. Relevant bands were excised, and extracted using the GeneJet gel extraction kit (Thermo Fisher Scientific), and Sanger sequencing was performed using the same primers.

**Table 1:** Primers used in PCR and Sanger sequencing reactions.

Gene	Name	Sequence (5’ to 3’)
*HBB*	Exon1F	GGATTACAAGGATGACGACGATA
*HBB*	Intron1R	tccttaaacctgtcttgtaacct
*HBB*	CrypticDonor1R	CACCAGCAGCCACCAACTT
*HBB*	CrypticDonor2R	CAGCAGCCTTGCCCCA
*HBB*	Exon1-2R	CACCAGCAGCCTGCCC
*HBB*	Exon2R	TCACTAAAGGCACCGAGCAC
*HBB*	Exon3R	AGCCACCACTTTCTGATAGGC
*NPT*	NeoF	GATGGATTGCACGCAGGTTC
*NPT*	NeoR	TCAGAGCAGCCGATTGTCTG

### RNA-Seq

RNA sequencing was performed by the Australian Genome Research Facility (AGRF) using Illumina Stranded Total RNA Prep with Ribo-Zero Plus rRNA Depletion yielding 150 bp paired-end reads sequenced to a depth of ~50 M reads per sample. Analysis was performed using a Snakemake [14] workflow integrating FastQC [15], Trimmomatic [16], STAR [17] and Samtools [18] to first adapter trim and align samples D94BP and Untransfected to a bespoke genome comprising Chlorocebus sabaeus (with endogenous *HBB* intact) and D94BP plasmid sequence appended as an additional chromosome. Negligible endogenous *HBB* coverage was observed in both D94BP and Untransfected. All samples were subsequently trimmed and aligned, with each sample aligned to a bespoke custom genome assembly comprising Chlorocebus sabaeus (with endogenous *HBB* masked) and sample specific plasmid sequence appended as an additional chromosome. Aligned reads and splice junctions generated during alignment were used to quantify transfected *HBB* intron 1 annotated splice junction utilisation and splice junctions utilising the two cryptic donors in exon 1.

Intron retention (IR) for transfected *HBB* Intron 1 was calculated by identifying the read depth of the first and last two bases of the annotated intron 1 splice junction and then averaging the four read depths to derive an *average intron read depth*. Then the read depth of last two bases of exon 1 and first two bases of exon 2 (adjoining intron 1) were identified and the average of these four read depths was used to derive an *average exon read depth*. Dividing the *average intron read depth* by *average exon read depth* provided a proxy for transfected *HBB* intron 1 IR. (See Table S1 for a list of samples, reference genome, bespoke genomes, alignment rate, and software versions).

### Statistical analysis

Statistical analysis and non-linear regression analysis were performed using the GraphPad Prism software.

## Results

### HBB intron 1 exhibits dominant but not exclusive usage of branchpoint at -37

**Fig. 1:**
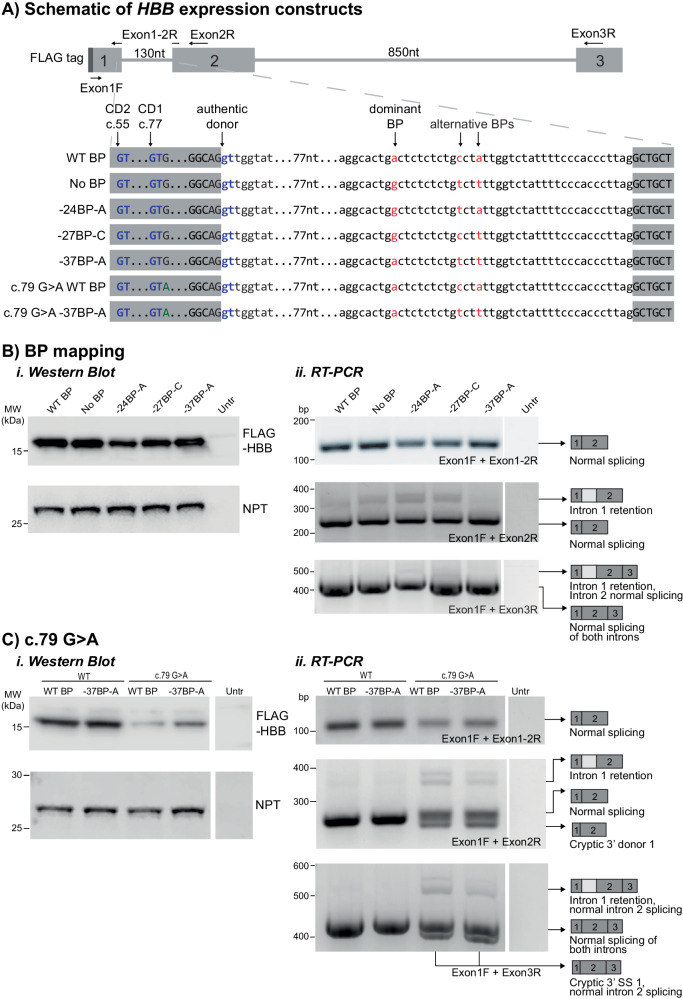
Mapping *HBB* intron 1 branchpoints. **A** Schematic of *HBB* minigene constructs subcloned into pCMV6, with N-terminal FLAG tag, three numbered exons, locations of primers used for RT-PCR, and pre-mRNA splicing-related features indicated. Exons are indicated in uppercase and introns in lowercase. Branchpoints (BP) shown in red, cryptic donor splice sites are shown in blue. Note that the exon 1-2 bridging junctional primer only binds to exon 1-2 spliced using the authentic splice site, and not the cryptic splice sites. **B**, **C** Transfection studies in COS-7 cells. Replicate plates were harvested simultaneously for ***i****.* western blot and ***ii****.* RT-PCR. 20 µg total protein was loaded per lane for western blots, membranes were probed with anti-FLAG antibody (HBB), and anti-neomycin phosphotransferase II antibody (NPT) as loading/transfection efficiency control. For RT-PCR, cDNA was reverse-transcribed from mRNA isolated from transfected COS-7 cells. The forward primer was positioned within the FLAG tag, and reverse primers bridging the exon 1-2 junction, or in exons 2 or 3. **B** Branchpoint mapping studies showing dominant but non-exclusive use of the -37-A branchpoint. **C** The c.79 G > A variant causes reduced protein expression, and increased intron retention, and is able to use th −37-A as the branchpoint for mRNA splicing.

We created a gene expression construct containing human *HBB* exons 1-3 with intervening introns, including a C-terminal FLAG tag (Fig. 1A) to enable in vitro manipulation of intron length and nucleotide composition. We utilised the machine learning branchpoint algorithm Branchpointer [10] to map potential branchpoint locations in *HBB* intron 1 and determine the nucleotide replacement at each location least likely to generate a useable branchpoint (lowest branchpoint probability score) (Figure S1). Based on these predictions, we generated branchpoint mapping *HBB* minigene constructs (Fig. 1A): −24BP-A, −27BP-C, and −37BP-A preserve the named predicted branchpoint and substitute all other predicted branchpoints with the nucleotide with the lowest Branchpointer probability score. The ‘no BP’ construct substitutes all predicted BP with the nucleotide with the lowest Branchpointer probability. Following transfection into COS-7 cells, all constructs generated protein of the correct molecular weight on western blot (Figure 1B*i*), with -24BP-A and -27BP-C expressing slightly lower levels. Concordantly, RT-PCR showed some canonical exon 1-2 splicing for all constructs, though with reduced normal splicing and increased intron 1 retention for −24BP-A and -27BP-C (Figure 1B*ii*). Our collective data identifies-37BP-A as the dominant branchpoint for *HBB* intron 1, aligning with previous studies [19]. Thus, we calculate D-BP length using the -37 dominant branchpoint, acknowledging that normal mRNA splicing and protein production from the ‘no BP’ construct suggests there is branchpoint redundancy in *HBB* intron 1.

We observed no difference in *HBB* exon 2-3 mRNA splicing between the branchpoint mapping constructs (Figure 1B*ii*) and so focussed all further studies of splicing changes to events occurring in intron 1 and between exons 1 and 2.

### Introduction of recurrent *HBB* c.79 G > A variant leads to increased cryptic splice site use

We generated further *HBB* minigene constructs bearing the c.79 G > A variant in exon 1 found commonly in β-thalassemia cases in Southeast Asia [8], with either the reference sequencing containing wildtype (WT) branchpoints, or with substitutions leaving only the dominant -37BP-A branchpoint (Fig. 1A). Transfection of these constructs into COS-7 cells revealed presence of the c.79 A variant severely reduces protein expression on western blot compared to WT c.79 G (Fig. 1C*i*). RT-PCR and Sanger sequencing showed the WT c.79 G construct shows exclusive canonical splicing via the authentic donor splice site while the c.79 A construct shows stochastic use of both the authentic donor splice site and upstream cryptic donor 1, as well as exhibiting increased intron 1 retention (Figure 1C*ii*). Inclusion of the c.79 A variant did not affect exon 2-3 mRNA splicing (Figure 1C*ii*).

### Shortening of *HBB* intron 1 D-BP distance puts pressure on spliceosome assembly, enhancing the use of upstream cryptic splice sites

**Fig. 2:**
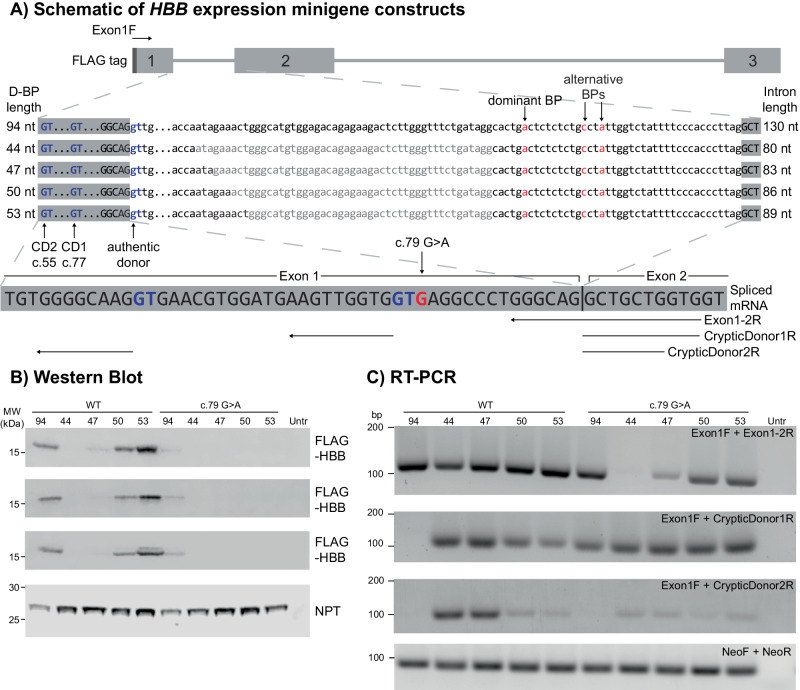
The effect of shortened donor to branchpoint distance in *HBB* intron 1. **A** Schematic of *HBB* minigene constructs subcloned into pCMV6, with N-terminal FLAG tag, three numbered exons, locations of primers used for RT-PCR, and pre-mRNA splicing-related features indicated. Exons are indicated in uppercase and introns in lowercase. Branchpoints (BP) are shown in red, cryptic donor splice sites are shown in blue. Wild-type and c.79 G > A versions of each plasmid were created for the full-length and deletion series constructs. Specific sequences deleted to reduce the donor-to-branchpoint (D-BP) distance are shown in grey, with constructs named to indicate the D-BP distance. Note that the exon 1-2 bridging junctional primer only binds to exon 1-2 spliced using the authentic splice site, while specific primers amplifying exon1-2 spliced using cryptic donors are named according to the corresponding cryptic donor. Transfection studies of *HBB* minigenes in COS-7 cells. Replicate plates were harvested simultaneously for **B** western blot and **C** RT-PCR. **B** 20 µg total protein was loaded per lane, membranes were probed with anti-FLAG antibody (HBB), and anti-neomycin phosphotransferase II antibody (NPT) as loading/transfection efficiency control. FLAG-HBB blots from 3 independent transfection experiments are shown. **C** cDNA was reverse-transcribed from mRNA isolated from transfected COS-7 cells. The forward primer was positioned within the FLAG tag, and reverse primers bridging the exon 1-2 junction from the authentic and cryptic donors as specified. One representative set of images is shown, from 3 independent transfection experiments.

To ascertain whether the minimum, spliceable, D-BP length (46 nt) for *EMD* intron 5 is recapitulated in a different genomic context, we generated a series of *HBB* gene constructs with intron 1 deletions that progressively shorten D-BP distance from the full length 94 nt, to 44–53 nt, with and without the exon 1 c.79 G > A variant (Fig. 2A). In silico predictions of pre-mRNA splicing and changes to canonical splicing for the full length 94 nt and shortened 44 nt constructs with both *HBB* c.79 G and c.79 A in SpliceAI [11] and BPP [12] showed that no additional splice sites were strengthened as a result of the changes (Table S2).

Following transfection of *HBB* intron 1 deletion minigenes constructs into COS-7 cells, no FLAG-HBB protein was detected on western blot from the WT c.79 G construct with a D-BP length of 44 nt. Incremental lengthening of D-BP length from 44 – 53 nt progressively restored protein expression (Fig. 2B). In contrast, only low levels of FLAG-HBB protein were detected with the full-length c.79 A construct, and no protein detected with any c.79 A 44 – 53 nt D-BP deletion construct (Fig. 2B).

RT-PCR for the exon 1-2 splice junction was performed using primers to specifically amplify canonical splicing from the authentic donor, or one of two upstream cryptic donor (CD) splice sites. Some canonical exon 1-2 splicing was detected for all WT c.79 G D-BP deletion constructs, however shortening the D-BP length resulted in activation of cryptic donor 1 (CD1, GT at c.77), with additional activation of cryptic donor 2 (CD2, GT at c.55) further upstream (Fig. 2C). The use of both cryptic donors decreased with lengthening of the D-BP distance in WT c.79 G constructs, with the sharpest decline in cryptic donor use between 47 and 50 nt (Fig. 2C). Variant c.79 A constructs showed elevated use of CD1 at all D-BP lengths, with a steep decline in canonical exon 1-2 splicing with reducing D-BP lengths and undetectable levels of canonical splicing with D-BP length of 44 nt (Fig. 2C). CD2 was not commonly activated for c.79 A D-BP deletion constructs and was not used at all for the full-length c.79 A construct likely due to the c.79 G > A substitution ( + 3 position from CD1) creating a stronger CD1 donor motif that outcompetes CD2 for spliceosomal binding. (Fig. 2C).

Importantly, the same pattern of authentic and cryptic donor use was observed using D-BP deletion constructs bearing only the dominant -37BP-A branchpoint (Figure S2).

### Pressure on spliceosome assembly remains for donor-to-branchpoint lengths of 53-56 nt

**Fig. 3:**
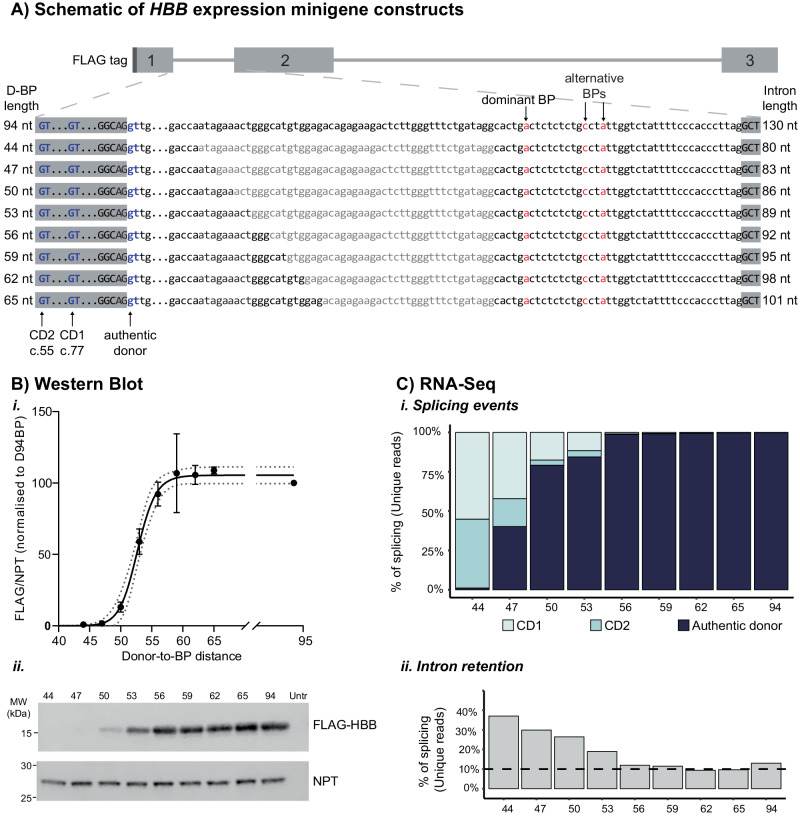
Western blot and RNA-Seq quantification of the effect of shortened donor to branchpoint distance in *HBB* intron 1. **A** Schematic of *HBB* minigene constructs subcloned into pCMV6, with N-terminal FLAG tag, three numbered exons, locations of primers used for RT-PCR, and pre-mRNA splicing-related features indicated. Exons are indicated in uppercase and introns in lowercase. Branchpoints (BP) are shown in red, cryptic donor splice sites are shown in blue. Wild-type and c.79 G > A versions of each plasmid were created for the full-length and deletion series constructs. Specific sequences deleted to reduce the D-BP distance are shown in grey, with constructs named to indicate the B-BP distance. *HBB* minigene constructs were transfected into COS-7 cells for **B** western blotting and **C** RNA-Seq analysis. **B*i*** Protein expression was quantified by western blot by normalizing FLAG (HBB) signal to neomycin phosphotransferase II (NPT) for transfection efficiency. FLAG/NPT signal at shortened D-BP lengths were normalized to that of the full-length D94BP minigene construct (3 biological replicates per construct, mean ± standard deviation) and plotted against donor-to-branchpoint distance using the non-linear regression model for specific binding with a Hill slope (Y=Bmax*X^h/(Kd^h + X^h), GraphPad Prism 8.2.1; R^2^ of 0.962^2^) (dotted lines represent the 95% confidence interval). Image from one representative blot is shown in **B*ii***, with 10 µg of total protein loaded per lane. **C*i*** Quantification of splicing at the *HBB* intron 1 cryptic and authentic donors as a percentage of all split-reads mapping to the *HBB* intron 1 authentic acceptor splice-site. **C*ii*** Quantification of intron 1 retention by calculating the average coverage of the first and last two bases of intron 1, and dividing it by the average coverage of the adjacent two exonic bases on either side.

Our collective evidence suggested that above the hard limit of biophysical space constraint for D-BP lengths of 44-47 nt exists a zone of intron lengths at which mis-splicing can still occur. Thus, we next sought to define which D-BP lengths are long enough to allow confident clinical interpretation of *no* mis-splicing risk. We generated additional *HBB* gene constructs with D-BP lengths from 44 to 65 nt (Fig. 3A), transfected them into COS-7 cells, quantified protein production by western blot (Fig. 3B) and splicing events by RNA-Sequencing (RNA-Seq, Fig. 3C & D).

Western blot of FLAG-HBB protein expressed from each construct was quantified, normalised to transfection efficiency using neomycin phosphotransferase (NPT) and plotted as a percentage of the full-length construct FLAG-HBB expression. Results from 3 separate transfection experiments were graphed using a non-linear regression model for specific binding, showing a steep, exponential decrease in protein production with reducing D-BP lengths within *HBB* intron 1 (Fig. 3B).

To assess the effect of reduced D-BP length on splicing, we performed whole transcriptome sequencing on COS-7 cells transfected with all WT c.79 G *HBB* D-BP constructs to quantify donor splice site usage (Fig. 3C*i*) and intron 1 retention (Fig. 3C*ii*). We aligned transcriptomic data from the nine constructs against D-BP length-specific custom reference genomes with endogenous *HBB* masked.

Taking all split reads mapping to the intron 1 acceptor, we quantified the relative proportion of events spliced using the authentic donor, CD1 or CD2. Aligning closely with protein data, we observe a steep, exponential decline in canonical splicing with decreasing D-BP lengths and increased use of the upstream CD1 and CD2 when the D-BP length is reduced below 56 nt with only trace canonical splicing at a D-BP length of 44 nt (Figure 3C*i*). Similarly, we saw a progressive increase in intron 1 retention as the D-BP length shortened, though D-BP lengths 56 nt and above exhibited similar levels of intron retention as the full-length construct (Figure 3C*ii*).

Applying similar retrospective analysis to data from our previous study [3] shows highly concordant specific exponential binding curves for *EMD* exon 5-6 splicing efficiency (Figure S3A) and emerin protein production (Figure S3B). These data constitute further evidence from another genomic loci that biophysical space constraint persists for lengths above the minimal D-BP 45 nt length threshold; in *EMD* intron 5 a D-BP length of 50 nt still resulted in only 50% normal exon 5-6 splicing and emerin protein production (Figure S3).

## Discussion

Spliceosome recognition and assembly on essential features of the intron is the crucial first step in pre-mRNA splicing, with lariat formation and excision critically dependent on D-BP length to enable spliceosomal A to B complex transition [3]. Our extended collective analyses of splicing defects due to D-BP deletions in *HBB* intron 1 and *EMD* intron 5 now concordantly indicate that: **1** Profound-to-complete splicing defects occur with D-BP lengths < 47 nt (i.e. ~90% impairment or greater); **2** Major splicing defects occur with D-BP lengths of 47–50 nt (i.e. ~50% impairment or greater; **3** Space constraint increases exponentially with D-BP lengths < 56 nt, with dramatically enhanced mis-splicing risk for genomic contexts where there is a local, competitive cryptic splice-site that becomes used in preference to the annotated splice-site to relieve space constraint pressure; **4** D-BP lengths of > 60 nt present low mis-splicing risk and can be considered benign. Importantly, our empirical evidence shows the precise level of splicing impairment at a given D-BP length can vary by one or two nucleotides between introns, indicating the influence of nucleotide composition and broader genomic context.

Intronic deletions reducing the donor-to-branchpoint length often fall outside of the canonical splice sites considered by many predictive algorithms used in clinical genetics and so their potentially deleterious effect on splicing is currently likely overlooked. Indeed, though our empirical testing shows profound-to-complete defects in *HBB* splicing with an intron 1 D-BP length of 44 nt, SpliceAI predicts no splicing abnormality. Prior to our own work, failure of splicing from short introns has been documented in *RECQL4* [20], though the underlying mechanism was not described. Informed by our previous study [3], a number of publications have considered shortened intron length when interpreting variants, including a pathogenic intron 31 *PKD1* variant [21]. Additionally, the recently developed tool Introme considers deletions rendering D-BP lengths < 45 nt as splice-altering [22]. While we agree D-BP lengths < 45 nt are near-guaranteed to alter splicing, our evidence now suggests this minimal length threshold will fail to detect many splice-altering, pathogenic, intronic deletions. Using Branchpointer to predict the dominant branchpoint in *RECQL4* intron 8 allows us to see that the variant described by Wang *et al* reduces the canonical donor-to-branchpoint distance from 59 nt to 48 nt in the proband, a length our *EMD* and *HBB* studies show is subject to spliceosomal space constraint providing a mechanism of pathogenicity.

Our studies suggest that donor selection in short introns is a balance between the intrinsic strength of available donor sites and the biophysical space constraint created by D-BP distance. Our full-length *HBB* gene construct (D-BP 94 nt) bearing the c.79 G > A variant shows increased relative use of the strengthened CD1 at c.77, in agreement with previous reports [9]. Further, we observe a profound switch toward predominant or exclusive use of CD1 in c.79 G > A intron 1 deletion constructs, indicating the threshold for near-complete mis-splicing due to D-BP space constraint can depend upon proximity of a usable cryptic splice-site. In contrast, for WT c.79G constructs, CD1 use increases incrementally with decreasing D-BP length, with additional upstream CD2 activation at the shortest D-BP lengths of 44 or 47 nt, indicative of acute space constraint pressures alone.

The use of either CD1 or CD2 in *HBB* exon 1 introduces a frameshift leading to the generation of a premature stop codon, critically reducing FLAG-HBB protein expression on western blot. The continued use of CD1 and CD2 beyond the critical 47 nt length limit for spliceosome assembly suggests some pressure on spliceosome assembly persists above this threshold. Indeed, our RNA-Seq analyses of *HBB* intron 1 show that at the mRNA level, this pressure impacts donor site selection at D-BP lengths up to 56 nt. Additionally, our western blot quantification suggests that a degree of variability in splicing and protein translation efficiency may persist up to D-BP lengths of 59 nt. Thus, while the presence of cryptic splice sites provides an additional layer of complexity for the interpretation of the effects of reduced D-BP distance our observations support the assignment of D-BP > 60 nt as a pragmatic threshold for splice-neutral outcomes.

It is important to acknowledge that precise calculations of D-BP length to determine biophysical space constraints for pre-mRNA splicing rely on precise definitions of both the donor and branchpoint; which for branchpoint can be ambiguous. A previous large-scale sequencing study of intron lariats identified more than one branchpoint in 47% of annotated introns, and that these introns were typically shorter than introns with only one branchpoint [10]. Although Branchpointer, trained on this sequencing data, has been shown to outperform other computational branchpoint prediction methods [23], the window of prediction is limited to 18–44 nt upstream of the acceptor [10]. While lariat sequencing of *HBB* intron 1 showed the usage of nucleotides other than those tested in our study, their use is comparatively rare compared to the usage of the dominant -37BP-A [19]. While the use of other branchpoints is retained upon ablation of -37BP-A, our identical western blot and RT-PCR results from a construct bearing only -37BP-A and a construct bearing all predicted branchpoints provide further support of -37 as the dominant branchpoint. Indeed, from all branchpoints we assessed, only the -37BP-A fully matched the yUnAy consensus branchpoint motif for human introns [24].

The use of in vitro gene construct assays is a common technique for studying pre-mRNA splicing, although there is no standardised protocol for their design. Informatic tools are extensively used to ensure that all cis-regulatory elements that may affect pre-mRNA splicing are included in the final construct. Gene construct design often involves shortening intronic regions to fit within construct size limits; we recommend design strategies carefully consider D-BP lengths to ensure efficient spliceosome assembly for modified introns. This is a particular concern for gene replacement therapy, where often the entire coding region of a gene is required to achieve therapeutic effect. The packaging capacity of commonly used adeno-associated vectors have traditionally been limited to <5 kb, so the reduction of intronic elements is a frequently used strategy. Our in vitro studies using the full *HBB* gene including all exons and introns and demonstrate that reducing D-BP distance has a serious impact on protein expression levels; just a few nucleotides difference in length can have a major impact on whether a protein is produced.

We employed RNA-seq to relatively quantify all splicing events arising from the *HBB* constructs overcoming the bias of targeted RT-PCR. While short-read RNA-seq enables agnostic detection of splicing outcomes, there were technical and informatic challenges using this approach for our *HBB* construct. *HBB* intron 1 retention or use of CD1 or CD2 encodes a premature termination codon that complies with nonsense-mediated decay; therefore, relative levels of annotated splicing (not targeted by nonsense-mediated decay) may be overestimated. Additionally, we could not express the construct in a cell line with robust endogenous *HBB* expression, as 150 nt short reads do not span the entire *HBB* mRNA and reads lacking the FLAG sequence could be attributed to the plasmid construct or genome. However, haemoglobin biosynthesis is specialised and not all cell lines, including HEK293 cells, with trace endogenous *HBB* were capable of synthesizing measurable levels of plasmid derived HBB protein. Overexpression models can also saturate transcription and translation systems causing accumulation of splicing intermediates. Finally, there were informatic challenges since the latest available annotation for COS-7 cells ChiSab1.1 (Ensembl release 109) [25] is based on orthologous proteins from vertebrate division of UniProtKB and may not cover the entire COS-7 transcriptome leading to potential low mapping rate (average 78.63%).

To overcome alignment issues, we created a custom genome based for each *HBB* deletion gene construct enabling efficient detection of identified splicing events. Despite all caveats of this RNA-seq approach, we observed striking concordance of deep analyses of D-BP lengths for two independent introns (*HBB* intron 1 and *EMD* intron 5), assessing splicing and protein data in combination, across different cells and tissues (skeletal muscle, primary myoblasts, COS7 and HEK293). The empirical reproducibility of splicing defects due to shortened D-BP lengths highlights the pressure exerted by biophysical space constraints.

Based on our collective evidence, we assert that intronic deletions that spare consensus splice motifs and render D-BP lengths > 60 nt are likely to be splice neutral while those $\le$ 60 nt have exponentially increasing risk of being splice altering, particularly in the presence of nearby cryptic splice-sites and splice altering variants. There is a high risk of splicing inefficiencies with D-BP lengths $\le$ 55 nt and a critical risk of profound or complete splicing defects with D-BP lengths $\le$ 50 nt. Due to the pathogenic potential of altered pre-mRNA splicing caused by reduced D-BP length, we urge careful consideration and interpretation of elements outside of consensus splice sites with more widespread inclusion in current in silico diagnostic prediction pipelines ≤56 nt should be closely interrogated, especially in the presence of nearby cryptic splice sites, while lengths >60 nt can be considered benign.

In addition to assisting variant interpretation in monogenic rare disorders and inherited cancer predisposition, our improved clarification of mis-splicing risk from D-BP deletions could hold relevance to somatic cancer genomics. Gene fusions are a common oncogenic basis for many cancers [26] and inherently involve the creation of a chimeric intron formed as part of the translocation. It is therefore important to consider the splicing motifs of the chimeric intron, particularly D-BP distance, as this could dramatically influence levels of the expressed oncogenic protein product.

### Supplementary information

Supplementary information

Supplementary Tables
