# Supplementary material for: Refining clinically relevant parameters for mis-splicing risk in shortened introns with donor-to-branchpoint space constraint
Source: Eur J Hum Genet. 2024 May 27;32(8):972–9. doi: 10.1038/s41431-024-01632-9 (PMC11291888; doi:10.1038/s41431-024-01632-9)
Supplement: Supplementary file 1 — Supplementary information [file 41431_2024_1632_MOESM1_ESM.pdf]

Supplementary Information

A) -24A>T (chr11:5248053T>A)

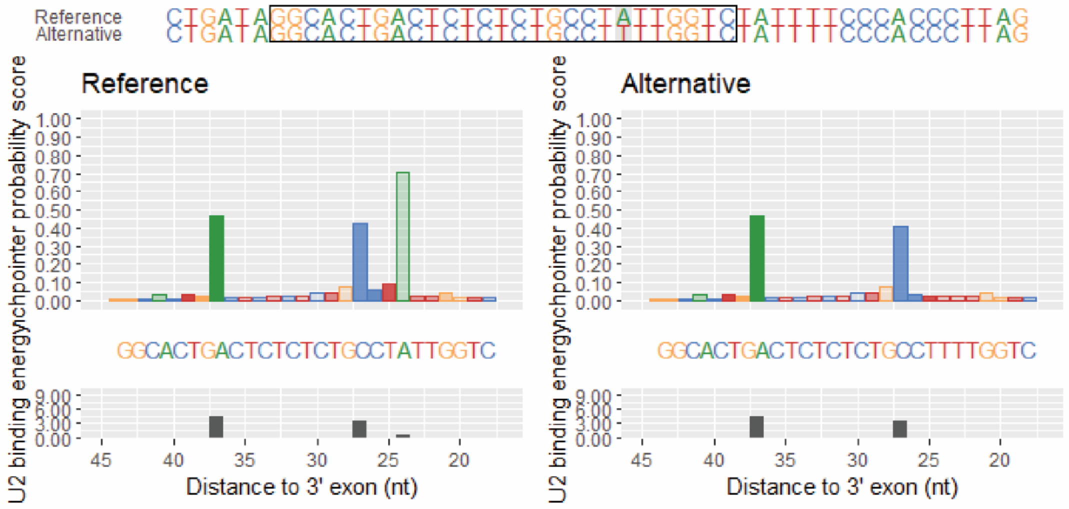

B) -27C>T (chr11:5248056G>A)

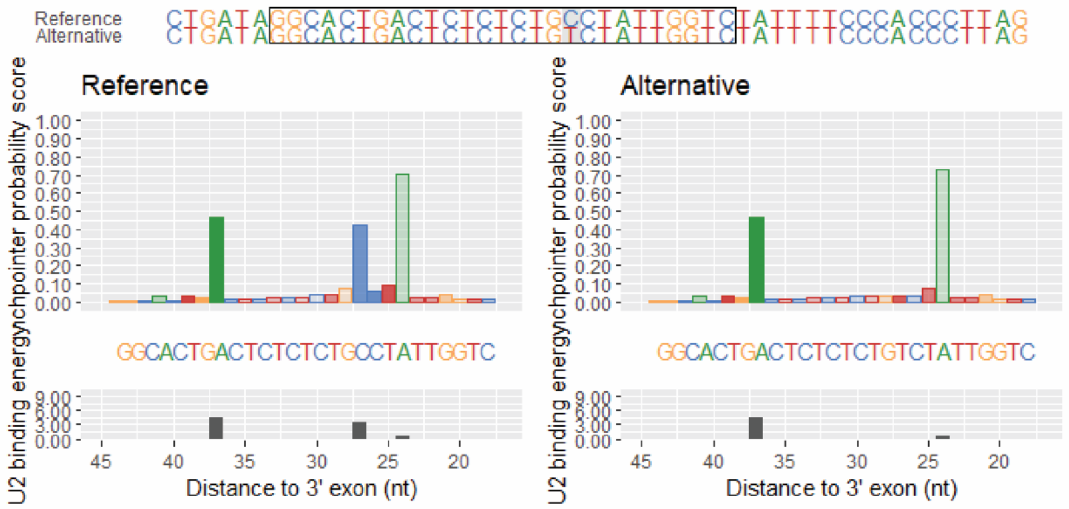

C) -37A>G (chr11:5248066T>C)

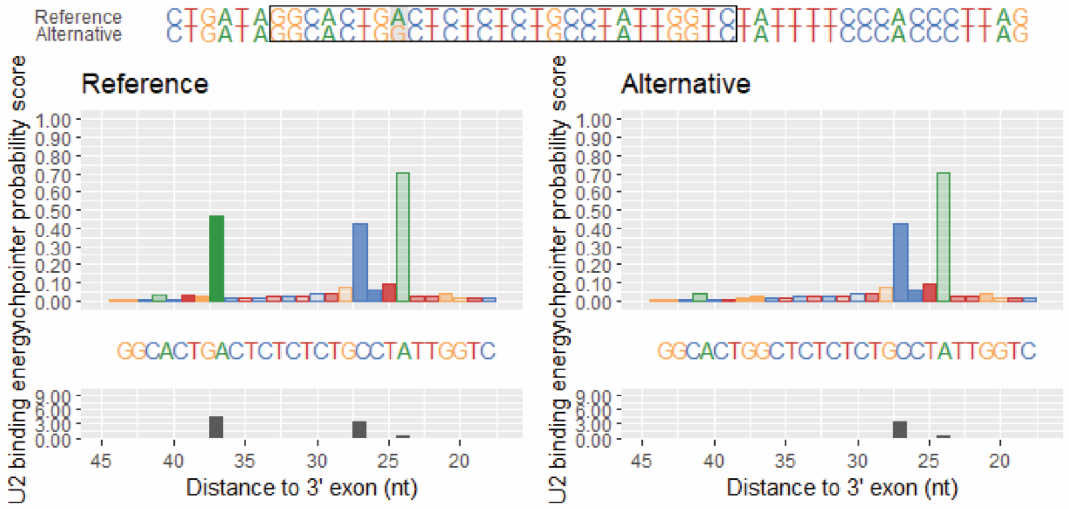

Figure S1: Branchpointer predictions of *HBB* intron 1 branchpoint nucleotide

Mapping the *HBB* intron 1 branchpoint location using the Branchpointer algorithm generated 3 potential branchpoint locations, at **A)** chr11:5248053T (-24), **B)** chr11:5248056G (-27) and **c)** chr11:5248066T (-37). Branchpointer also predicted the nucleotide change at each of these locations that gave the lowest Branchpointer probability score, and all changes also resulted in the complete elimination of U2 binding energy.

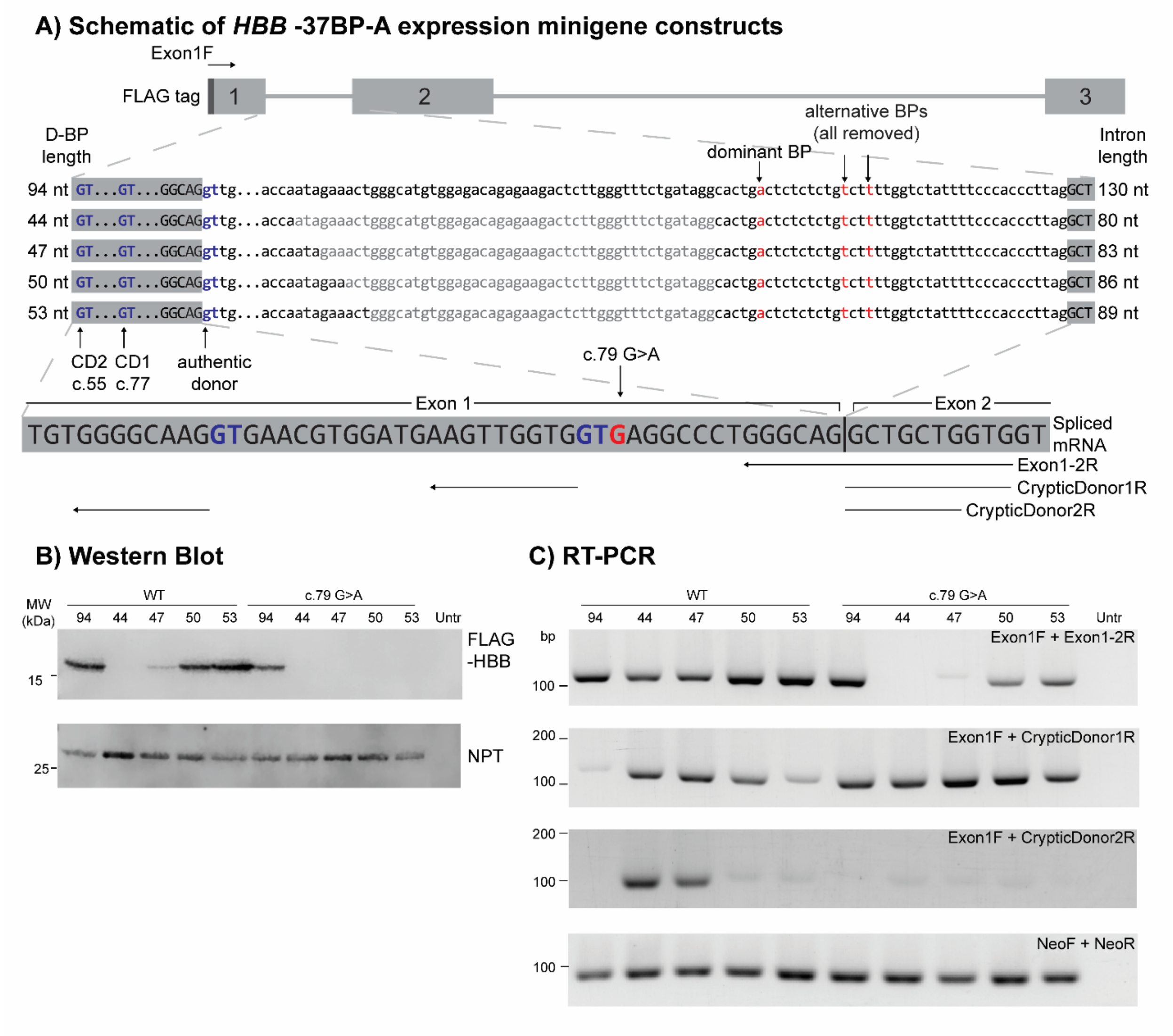

Figure S2: The effect of shortened donor to branchpoint distance in *HBB* intron 1 with -37BP-A as the only branchpoint present

A) Schematic of *HBB* minigene constructs subcloned into pCMV6, with N-terminal FLAG tag, three numbered exons, locations of primers used for RT-PCR, and pre-mRNA splicing-related features indicated. Exons are indicated in uppercase and introns in lowercase. Branchpoints (BP) shown in red, cryptic donor splice sites are shown in blue. Wild-type and c.79G>A versions of each plasmid were created for the full length and deletion series constructs. Specific sequences deleted to reduce the donor-to-branchpoint distance are shown in light grey, with constructs named to indicate the donor-to-branchpoint distance. The exon 1-2 bridging junctional primer only binds to exon 1-2 spliced using the authentic splice site, while specific primers amplifying exon1-2 spliced using cryptic

donors are named according to the corresponding cryptic donor. **B and C)** Transfection studies in COS-7 cells. Replicate plates were harvested simultaneously for **B)** western blot and **C)** RT-PCR. 20 µg total protein was loaded per lane for western blots, membranes were probed with anti-FLAG antibody (HBB), and anti-neomycin phosphotransferase II antibody (NPT) as loading/transfection efficiency control. For RT-PCR, cDNA was reverse-transcribed from mRNA isolated from transfected COS-7 cells. The forward primer was positioned within the FLAG tag, and reverse primers bridging the exon 1-2 junction from the authentic and cryptic donors as specified. Images shown are representative of 3 independent transfection experiments.

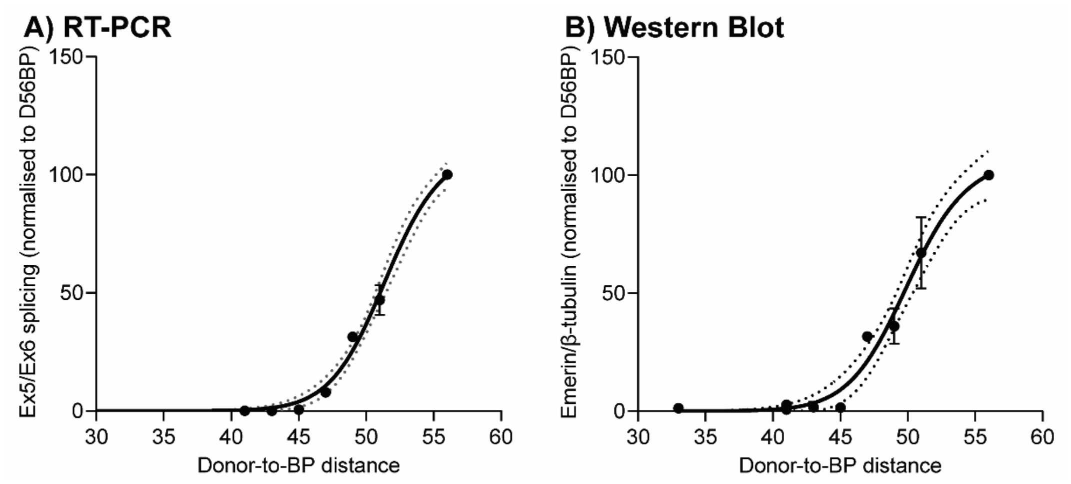

Figure S3: Reanalysis of EMD data as supportive of HBB data

Analysis of our previous data (3) from a series of EMD minigene constructs with progressively shortened intron 5 D-BP lengths assessed by **A)** RT-PCR and **B)** western blot. Expression signal at shortened D-BP lengths were normalized to that of the full length D56BP minigene construct (2 biological replicates per construct, mean ± standard deviation) and plotted against D-BP distance using the non-linear regression model for specific binding with a Hill slope (Y=Bmax*X^h/(Kd^h + X^h), GraphPad Prism 8.2.1; R2 of 0.9906 for **A)** and 0.966 for **B)**). Dotted lines represent the 95% confidence interval.
